# Fear Similarly Alters Perceptual Estimates of and Actions over Gaps

**DOI:** 10.1371/journal.pone.0158610

**Published:** 2016-07-07

**Authors:** Michael N. Geuss, Michael J. McCardell, Jeanine K. Stefanucci

**Affiliations:** 1 Max Planck Institute for Biological Cybernetics, Tübingen, Germany; 2 University of Utah, Department of Psychology, Salt Lake City, United States of America; VU University Amsterdam, NETHERLANDS

## Abstract

Previous research has demonstrated an influence of one’s emotional state on estimates of spatial layout. For example, estimates of heights are larger when the viewer is someone typically afraid of heights (trait fear) or someone who, in the moment, is experiencing elevated levels of fear (state fear). Embodied perception theories have suggested that such a change in perception occurs in order to alter future actions in a manner that reduces the likelihood of injury. However, other work has argued that when acting, it is important to have access to an accurate perception of space and that a change in conscious perception does not necessitate a change in action. No one has yet investigated emotional state, perceptual estimates, and action performance in a single paradigm. The goal of the current paper was to investigate whether fear influences perceptual estimates and action measures similarly or in a dissociable manner. In the current work, participants either estimated gap widths (Experiment 1) or were asked to step over gaps (Experiment 2) in a virtual environment. To induce fear, the gaps were placed at various heights up to 15 meters. Results showed an increase in gap width estimates as participants indicated experiencing more fear. The increase in gap estimates was mirrored in participants’ stepping behavior in Experiment 2; participants stepped over fewer gaps when experiencing higher state and trait fear and, when participants actually stepped, they stepped farther over gap widths when experiencing more fear. The magnitude of the influence of fear on both perception and action were also remarkably similar (5.3 and 3.9 cm, respectively). These results lend support to embodied perception claims by demonstrating an influence on action of a similar magnitude as seen on estimates of gap widths.

## Introduction

### Fear similarly alters perceptual estimates of and actions over gaps

Embodied approaches to perception argue that visual angles specifying spatial layout (heights, distances, etc.) need to be scaled in order to have meaning to an observer [1]. Specifically, some have argued that visual angles are scaled by the size of one’s body or one’s emotional state because these scales are related to the costs associated with acting [1–3]. Actors learn to interpret visual information through acting in the environment, and these perceptual processes have been shaped through evolutionary history [3,4]. Empirical findings in support of these claims have demonstrated, for example, that hills are estimated as steeper when exhausted [5], gap-widths are estimated as smaller when the body is made larger [6], and heights are estimated as taller when afraid [7, 8].

The purported purpose of these changes in perception is to alter future actions in a manner that reduces the likelihood of injury [6,9]. Given a strong link between perception and action, biases in estimates of heights when afraid, for example, directly inform the actor of the costs associated with acting by altering *perception* of spatial properties. According to this argument, actors should then behave as if heights are taller or act more conservatively around heights in order to be safe. Whether this occurs, however, is unknown and there is some evidence to suggest that a similar change in action, as on estimates of spatial properties when afraid, may not be found [10]. Specifically, the two-visual streams hypothesis suggests that visual information is processed differently depending on the type of task (for action or identification), suggesting that an influence of fear on perceptual estimates may not necessitate a change in action [11, 10]. Further, others have argued that it is important for visual information to result in an accurate model of space to effectively guide actions [12, 13].

The goal of the current studies was to test whether there is a similar influence of fear on estimates of spatial layout as on visually guided actions. Visually guided actions are actions carried out on the basis of visual information [14]. To test this, we investigated whether fear altered stepping over gaps in the same direction, and to the same magnitude, as perceptual estimates of the width of gaps placed above different heights. For example, if the perception of the width of a gap presented over a height is altered by fear, do people change how they step over the gap? More importantly, do they do so in a manner consistent with the potential perceptual bias of the gap’s width?

To motivate this work, we will first introduce research that has found an influence of fear on perceptual estimates of real world heights and arguments for why such perceptual biases might affect performance of actions around heights, which would support embodied perception accounts. We will then discuss research that has demonstrated dissociations between action and perception measures, which suggest that an effect of fear on perceptual estimates may not necessarily indicate a change in the performance of visually guided actions. Lastly, we will provide an overview of the current studies to motivate the specific methodology used to investigate this open theoretical question.

### Effects of fear on height perception

Heights are estimated as taller when more afraid [9,15–17]. Fear can arise due to two different sources, which are not mutually exclusive: state and trait fear. State fear is defined as temporary situational changes in one’s level of fear, whereas trait fear is defined as long-term, persistent and more generalized fear that can differ across individuals [18,19]. Both sources of fear have been shown to increase estimates of heights, although the influence of state fear on estimates of heights is not as reliably observed as trait fear. For trait fear, estimates of heights are greater for participants who have high trait fear compared to those with low trait fear [17]. Trait level fear is often assessed using the Acrophobia Questionnaire (AQ), which asks participants to rate how scared they would be in several height-related scenarios [20]. In non-height-phobic samples, there are mixed results as to whether estimates of heights are influenced by state fear [8,16]. For example, estimates of height were positively correlated with self-reported levels of fear in one but not another experiment in Stefanucci and Proffitt [8] where, in both experiments, fear was induced by viewing the judged height. Additionally, state fear was not found to influence height estimates in a recent study on the influence of texture on height perception [21]. Estimates of heights, however, were increased when participants were asked to visualize falling from a height [22], after they viewed physically arousing images [16], or when the consequences of falling from a short height were increased by placing a bed of nails at the base of the height, thereby increasing its danger from falling [15]. These manipulations were intended to increase state levels of fear. In addition, when danger is removed from the situation by having participants estimate the height from below or by asking participants to estimate extents on a horizontal ground plane, the influence of fear on perceptual estimates disappears [16,23]. These results suggest that state fear may influence estimates of spatial properties when there are potentially dangerous consequences for acting on the extent. However, the overall mixed results suggest that additional investigations of state fear on perceptual measures is warranted to understand the limitations of these effects.

### Influences of emotion on action

While previous work has posited a connection between changes in perceptual estimates and future actions, no singular paradigm has tested changes in perception and action as a function of both state and trait fear and their potential interaction. Related work, however, has found that anxiety may influence the selection and performance of actions, suggesting that an influence of fear on actions could result from a change in estimates of spatial layout when afraid [24–26]. Specifically, anxiety has been shown to influence affordance judgments—judgments about whether an action is possible or not given the relationship between one’s capabilities and environmental constraints [25–27]. Bootsma et al. [25] investigated the influence of anxiety on judgments of whether targets were reachable. They found that the point at which participants judged targets to be within reach was not altered by anxiety, but the consistency of those responses was affected. Anxious participants’ responses were more inconsistent than controls. Graydon et al. [26] investigated the influence of anxiety on judgments of reaching, grasping, and passing one’s hand through holes. Unlike Bootsma et al. [25], they found that anxious participants judged that they were less capable of performing all actions when compared to non-anxious controls. In addition, it is well known that in high-anxiety situations, one can “choke under pressure,” or execute a task significantly worse than when not anxious [24]. Changes in the performance of actions, and not just the decision to perform an action, when anxious have been demonstrated for multiple actions including batting, shooting a basketball, skiing, and climbing a wall [28–32]. While anxiety differs from fear in terms of the immediacy of threat [33], the influence of anxiety on affordance judgments and performance of actions suggests that fear may influence whether an action is performed and how the action is executed.

In work related to the current studies, Jiang and Mark [27] investigated whether affordance judgments for stepping over gap widths would change due to the depth of the pit underneath the gap. They found that, on average, participants were conservative in their judgments while standing on the ground, indicating that they could only, on average, step across gaps that were 0.60 times the participants’ eye heights. Interestingly, they found that as the depth of the pit increased, participants’ judgments of what the environment afforded became more conservative. Jiang and Mark [27] speculated that these results could have been due to an increase in fear at higher heights but they did not directly measure changes in fear. In addition, participants only judged whether an action was possible and did not actually act or estimate the size of the gap width. In separate work, fear has also been shown to influence judgments of whether a sound emitting object was within reach [34]. Specifically, participants who were more afraid indicated sounds that originated from farther away to be within reach compared to participants who were not scared, suggesting that fear decreased perceived distance. This effect, however, disappeared when participants were allowed to see the object. Previous work on the influence of fear on judgments of action possibilities suggests an influence of fear on actions may be possible, but it remains unclear if the magnitude of these changes is related to changes in perceptual estimates, such as a change in the estimation of the gap width itself due to fear.

### Dissociations of perceptual estimates and visually guided actions

Whereas embodied perception accounts argue that a change in perception when afraid should alter subsequent actions, a change in perceptual measures when afraid may not necessarily lead to a change in action. Milner and Goodale [10] argued that visual information is processed differently depending on whether the goal is to visually guide an action or to consciously make a perceptual estimate, suggesting that an influence of fear on perceptual estimates may not necessarily affect actions [35]. Using pictorial illusions, multiple studies have found dissociations between estimates of a stimulus and actions directed toward that stimulus [36] (for review see [11, 37]). For example, in the Ebbinghaus illusion, two same-size discs are perceived to be different sizes depending on the relative size of circles placed around each disc. When participants are asked to match the size of the discs using a reference extent (distance between finger and thumb) or indicate which disc is larger, judgments are biased by the illusion [36,38]. However, if participants are asked to reach toward the target, the grip aperture corresponds to the actual size of the disc [36].

Similar dissociations between perception and action have been found when performing larger-scale actions, such as a walkable version of the Müller-Lyer illusion [39,37,40.^41^]. In this illusion, a straight line is presented with a hoop either placed overlapping with the line itself or at the endpoint of the line with no overlap. Participants were either asked to estimate the length of the line by using a visual matching task, where they adjusted a reference extent to match the viewed extent, or to walk without vision to the endpoint of the line [41]. Visually matched estimates were biased by the placement of the hoop but the performance of the action was not. Cañal-Bruland et al. [40] tested whether the dissociation between measures depended on egocentric viewing of the stimuli. When participants stood at the base of the Müller-Lyer illusion, a similar dissociation was found between verbal estimates and tossing a beanbag such that it landed near a line [40]. Interestingly, the dissociation between measures disappeared when participants viewed the stimuli from farther away and thus had greater access to allocentric information. These results suggest that viewing from an egocentric perspective may privilege actions to accurate perceptions of spatial layout.

A similar dissociation between perception and action measures is also present in more ecological environments. For example, distances are estimated as shorter and hills as steeper than they really are, yet these biases in awareness do not typically influence the performance of actions on these environmental features [5]. Participants were asked to estimate the slant of a hill by verbally reporting the slant in degrees, adjust a disc to equal the perceived cross-section of the hill (matching task), and tilting a board to match the slant of the hill with an unseen hand (haptic task) [5]. Results showed the slant of the hill to be vastly overestimated with verbal reports and matching tasks, but the haptic tasks were more accurate. These results were interpreted to indicate that action-based measures have privileged access to an accurate perception of the slant of the hill as to allow for appropriate stepping behavior. However, the validity of this dissociation has been debated based on issues of whether using haptic palm boards are an appropriate measure of actions [42].

### Overview of Current Experiments

It is an open question as to whether fear (both state and/or trait) influences the performance of actions to the same extent as it does estimates of space. It is possible that the influence of fear is restricted to conscious perception, given the prior work on visual illusions and dissociations between perception and action. In the current set of studies, we investigated whether fear influenced the performance of actions in the same direction and to the same magnitude, as changes in perceptual estimates. The number of practical actions, however, that can be performed on or around heights is relatively small. One possible action that could be performed is stepping over a gap suspended above a height. Thus, the current experiments tested whether the perception of gap-widths (Experiment 1) and the performance of stepping over gap-widths (Experiment 2) were altered by one’s state and trait fear when viewing gaps at varying heights. Separate groups of individuals estimated gap-widths or stepped over gap-widths to reduce a potential task demand of participants intuiting a relationship between estimates and stepping and then consciously biasing their responses or actions to coincide with their other response. Admittedly, there is the potential for participants to intuit a relationship between height or fear and each response measure. However, because measures of fear can vary within a person, the subjective meaning of an emotional measure may vary between people, the current studies focused on *changes* in state fear within individuals in response to viewing various heights. We believe change in state fear is a better measure than single, discrete reports and may additionally shed light on the effect of state fear on perceptual estimates which, in previous research, is sometimes evident and sometimes not. Trait levels of fear were also assessed given previous research demonstrating that changes in perceptual estimates may occur through either short-term (state) or long-term (trait) mechanisms. Both experiments were conducted within a virtual environment to allow participants to see multiple gap widths at different heights in quick succession, to measure changes in state fear over time, and to have participants perform actions over dangerous heights without placing them in any real danger.

In addition to testing whether an “act-on-able” spatial dimension, such as a gap width, is influenced by fear, Experiment 1 assessed the novel question of whether perception of another spatial property (e.g., a gap) other than the extent of heights was affected by fear. Experiment 2 investigated whether performance of action was changed when afraid. We tested the influence of fear on stepping over gaps both in terms of whether participants performed the action and, if they stepped over the gap, how they stepped. If participants stepped, we measured how far over the gap they stepped. We hypothesized that if changes in perception of spatial layout serve the purpose of altering actions, as claimed by the embodied perception approach, then stepping over the gap would be altered by fear in the same direction and magnitude as estimates of gap widths.

## Experiment 1: Estimates of Gap Widths

In Experiment 1, estimates of gap-widths, extending in depth on a horizontal plane, were assessed in order to determine whether a fear of heights influenced a dimension of space in the vicinity of the height. In addition, these perceptual estimates were of a spatial dimension that could be acted upon at the height. Participants visually matched the distance between two platforms. The platforms were placed either on the ground, 3 m above the ground, or 15 m above the ground. The height of the gaps was altered to induce fear. Trait fear was indexed with the Acrophobia Questionnaire (AQ) after all trials were completed [20]. In addition, participants subjectively rated their levels of state fear after viewing each height using the Subjective Units of Distress Scale (SUDS). If the influence of fear on space perception extends to dimensions of space beyond just heights, then we expected estimates of gap widths to be larger for participants who indicated being more afraid.

### Method

#### Participants

Thirty-six (24 female, 12 male) University of Utah students with a mean eye-height of 1.58 m (*sd* = .13) participated in the experiment for course credit. All participants were naïve to the purpose of the experiment, gave written informed consent, had normal or corrected-to-normal vision, and showed no deficit in stereo vision. The experimental procedure was approved by the institutional review board at the University of Utah and was in accordance with the Declaration of Helsinki.

#### Apparatus

**Fig 1:**
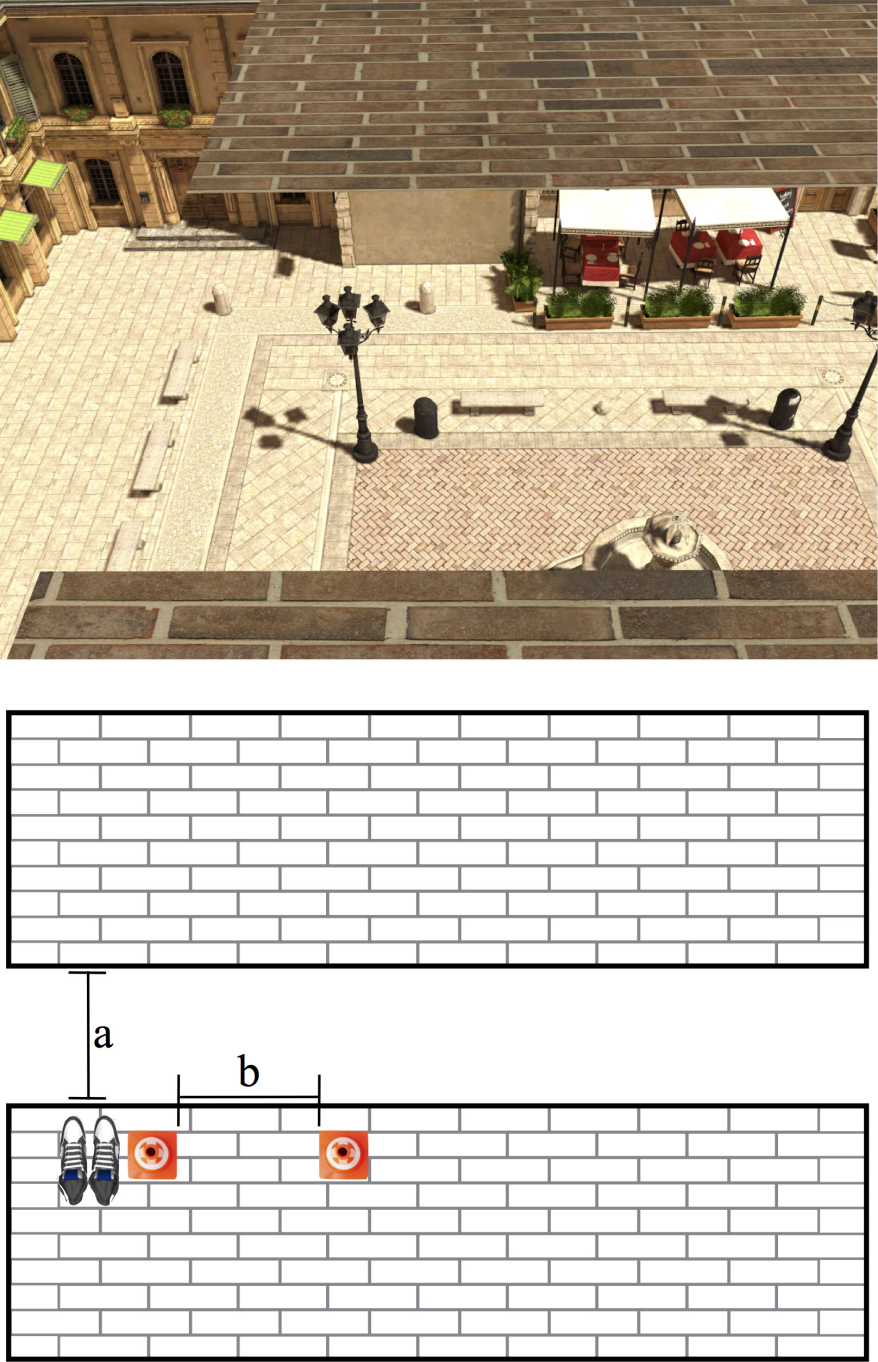
Virtual Environment. Screenshot of the virtual environment viewed from the participant’s standing location and schematic drawing of the estimation task (not-to-scale). Participants began the trials standing on the near brick platform and were tasked with estimating the distance from the edge of the near brick platform to the edge of the far brick platform (a) by adjusting the distance between two cones placed to the right of the participant (b). Participants only stepped across the gap in Experiment 2.

The virtual environment was displayed in stereo using an NVIS nVisor SX 60 head-mounted display (HMD) with a resolution of 1280 x 1024 pixels in each eye and a 42° x 34° field of view. Inter-pupillary distance was set for each participant. The location of the participant was tracked using an 8 camera PPT-H tracking system. Participants viewed the virtual environment from a virtual platform (45 m long, 2 m wide, 5 cm tall) that was placed at different heights within the virtual environment. A second platform of the same proportions was placed at various distances from the platform on which participants stood, creating multiple gap widths. The virtual environment was a model of a piazza provided by Vizard and included rich textures, objects, and lighting. Fig 1 shows the two platforms that were presented in the virtual environment. In addition, the location of participants’ feet was tracked using two PPT infrared markers per foot (a marker was placed on the toe and heel of each foot). These markers were used to portray virtual feet to the participants. The size of the virtual feet was scaled to match participants’ real foot sizes as previous research indicated foot size can be used to alter perception of near distances [43].

Participants adjusted the location of a virtual cone (20 cm tall, 10 cm radius at the base) along the length of the platform to perform a visual matching task in order to indicate perceived gap width. Participants moved the cone by pressing one of two buttons via a Logitech X-Box controller. If the participant held the button down, the cone would accelerate at a rate of 0.01 m/s^2^ and had a max speed of 20 m/s. To measure trait levels of fear, participants filled out the Acrophobia Questionnaire [20] at the end of the experiment. Participants also filled out a short debriefing questionnaire (4 questions) that assessed whether they intuited the hypotheses of the experiment.

#### Design

All participants viewed 4 gap widths (.45 m to 1.5 m at .35 m intervals) at 3 heights (0 m, 3 m, and 15 m) in blocks of trials. At each height, all widths were presented before moving to the next height, which constituted one block of trials. Within the block, the gap widths were displayed in either increasing order or decreasing order (i.e., participants saw the .45 m increasing to the 1.5 m width in succession, or they saw the 1.5 m width to the .45 m width in succession). Heights were randomly presented throughout the experiment with the exception that the 0 m height was always first and last. The 0 m height was presented first to allow participants practice with the controller and to provide estimates of gap widths before any fear was induced due to standing at higher heights. The experiment included, in total, 32 trials across all blocks.

#### Procedure

After completing the informed consent, participants were told that on a given trial, they would see a blank screen followed by the virtual environment. Their task was to look down and make three judgments all on a scale of 0–100 (how likely they were to fall if they attempted the step, how hurt they would be if they fell, and how afraid they were of the height), with 0 indicating the least and 100 the most. These ratings were included in order to determine whether changes in perceptual estimates were due to general fear (SUDS), a specific consequence of falling (Hurt), or the perceived likelihood of falling. Following the questions, participants were instructed to adjust the location of a virtual cone so that the distance between the near edge of the moveable cone and a cone located next to their feet equaled the distance from the near platform to the edge of the far platform. The cones were positioned 90 degrees to the right of the participants on the virtual platform on which they stood. The initial position of the cone was randomized and counter balanced within-participants so that it started next to the participants’ feet on half of the trials and at 2 m from their feet on the other half of trials. Participants were permitted to look back and forth between the gaps and the cones as often as they needed to be as accurate as possible. No feedback about the accuracy of their estimates was provided. At no time before or during the experiment were participants allowed to simulate stepping over the gap with their virtual feet. After completing the gap estimates, participants removed the HMD. They then completed the AQ, the debriefing questionnaire, and demographic information was recorded. In the debriefing questionnaire, participants were asked to indicate what they believed the hypothesis and manipulations of the study to be. Only five out of 36 participants correctly identified the hypothesis. Two participants identified the correct variables but indicated the opposite relationship and three participants indicated the correct variables but no relationship. The rest indicated an influence of height or gap width on fear, an inquiry into accuracy of judgments, or indicated that they did not know. The raw responses from each participant are available to view in the data accessible online. The entire experiment lasted about 45 minutes.

### Results

#### Mean Estimates

Descriptive statistics are present in Table 1. Specifically, estimates were averaged across participants to obtain the mean estimate (and SD in estimate) per gap width at each height. It should be noted that these means do not take into account any differences in fear across or within individuals. Participants, on average, overestimated gap widths.

**Table 1:** Mean Estimates.

	0 m	3 m	15 m
.45 m	.706 (.167)	.737 (.233)	.749 (.219)
.80 m	1.071 (.149)	1.112 (.165)	1.136 (.192)
1.15 m	1.417 (.221)	1.453 (.219)	1.506 (.232)
1.50 m	1.817 (.281)	1.811 (.308)	1.878 (.313)

Mean (SD) estimates for each gap width (rows) for each height (column) are displayed in meters. Participants overestimated all gap widths and this overestimation increased with height.

#### Measures of Fear

SUDS scores were entered for each trial and were person-centered so that effects of SUDS scores are interpreted as changes in self-reported levels of fear from individual normative levels of fear. On average, participants had a positive reaction to viewing the heights of 31.3 (SD = 31.09), with a range from 0 to 100. Acrophobia scores were 35.68 (SD = 21.65) on average, with a range from 4 to 88. AQ score was grand-mean centered. Therefore, any effect of AQ score should be interpreted relative to the average level of trait fear within the sampled population.

Because each participant completed the three subjective ratings (SUDS, chance of falling, and injury from falling) at each combination of height and gap width, we conducted correlations between these three responses for each participant separately. The strength of these correlations indicates how consistent participants’ ratings were across environmental conditions. SUDS and chance of falling were highly correlated for 32 out of 36 participants (all significant *rs* greater than .59, all *ps* < .001). SUDS and perceived injury from falling were significantly correlated for 15 out of 36 participants (all significant *rs* greater than .39, all *ps* < .03). Chance of falling and perceived injury from falling were significantly correlated for 24 out of 36 participants (all significant *rs* greater than .39, all *ps* < .03). Because of high correlations between these measures for a large portion of the participants and the emphasis of this paper on assessing state level fear, all reported analyses focused on ratings of subjective units of distress (i.e., fear), which was our main variable of interest.

### Are gap estimates predicted by fear?

A multi-level regression analysis was conducted using R v 0.98 and the lme4 package. Gap-Width Estimates, in meters, were regressed onto SUDS scores (person-centered; original scale 0–100), Gap-Width (scaled by participant’s eye-height and person centered), Heights (centered at 3 m and entered in meter units), and onto Trait Level Fear (grand-mean centered; sample range from 4–88). All factors were allowed to interact. Therefore, all effects follow normal regression interpretation and should be interpreted with respect to the centered value of all other variables. Scaling the gap width to each participant’s eye-height normalized the gap widths across participants with different stepping ability (*m* = .624, *sd* = .26, *range*: .26 to 1.04). A one unit increase in eye-height scaled gap width is the equivalent of extending the gap width by 100% of a participant’s eye-height. All betas reported are unstandardized. As expected, larger gaps were estimated to be larger than smaller gaps (β = 1.65, *p* < .001). Estimates of gap widths were also larger at higher heights than lower heights (β = 0.0046, *p* = .042). For every one meter increase in height, the model estimated a .0046 m increase in gap estimates.

**Fig 2:**
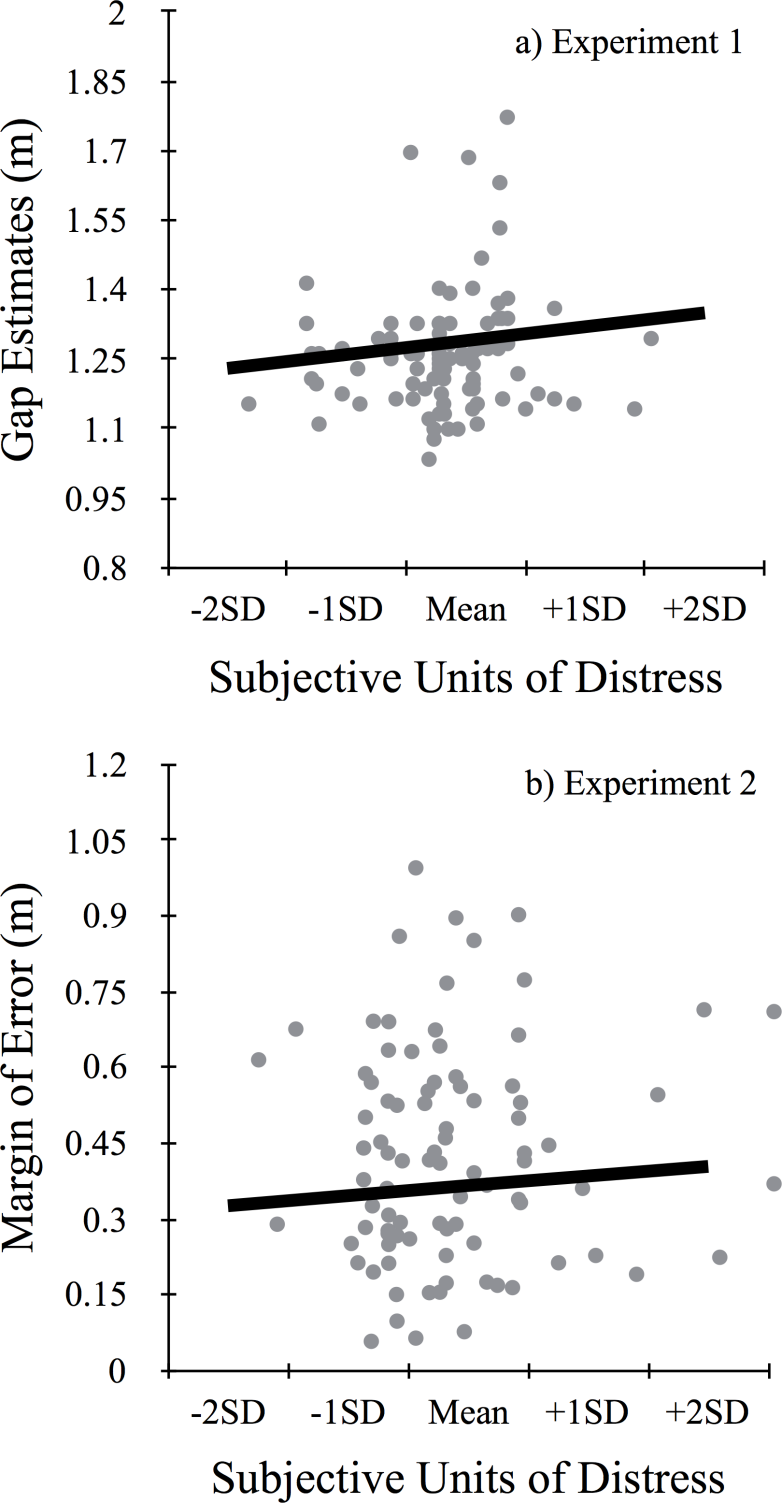
Estimates and Action as a Function of State Fear. (a) Estimates of gap width from Experiment 1 and (b) margin of error, or how far over the gap people stepped, from Experiment 2 are plotted as a function of state levels of fear (mean, +/1 SD) as measured by the Subjective Units of Distress Scale (SUDS). Both estimates of gap widths and margin of error increased with state fear and by a similar magnitude (5.3 and 3.9 cm, respectively).

After controlling for all other factors in the model, there was a significant main effect of SUDS scores (β = 0.0008, *p* = .016; see Fig 2A). For every one unit increase in SUDS scores, the model estimated a .0008 m increase in gap estimates. To understand this effect better, we computed the predicted gap-width estimate when participants reported being less and more afraid. Estimates of gap widths were greater on trials for which participants reported feeling more afraid compared to trials when they felt less afraid. On trials where participants reported being less afraid (-1 SD, SUDS = 0.02), participants estimated the gap width to be 1.26 m, on average. On trials where participants reported being more afraid (+1 SD, SUDS = 62.39), participants estimated the gap width to be 1.32 m, on average. Thus, we saw an increase in perceptual estimates of gap width by 5.3 cm, on average, as a function of within-person changes in state fear. There was no influence of trait fear on estimates of gap widths (*p* = .39).

The results demonstrated that perceptual estimates of gap widths increased as participants reported experiencing more fear. This finding is important because it shows that feelings of fear can influence estimates of space that are not particularly threatening in and of themselves. In addition, the size of the change is significant (~ 5 cm) when considering the action of stepping over. Given the consistent influence of trait fear on estimates of heights in previous work, we expected that there would be an influence of trait fear on estimates of gap widths placed above heights. The non-significant influence of trait fear on gap-width estimates, however, could have been due to less attention heeded to the height itself since the task required estimating gaps. In the next experiment, we investigated whether changes in actions were altered as a function of one’s level of fear as well.

## Experiment 2: Stepping over Gap Widths

The goal of this experiment was to determine whether actions are influenced by fear. In Experiment 1, estimates of gap widths displayed at various heights increased as state levels of fear increased. In the current experiment, participants were asked to step over the gaps presented at various heights from Experiment 1. If participants stated that they believed they could successfully step over the gap, then they executed the step. Performance was indexed in terms of whether or not the gaps were perceived as step-over-able and if so, how far onto the next platform participants stepped (margin of error). Fear was measured in the same manner as Experiment 1—SUDS were used to index state level fear and AQ to index trait level fear. If changes in estimates of spatial properties when afraid are relevant to the performance of actions, participants should either opt to step over fewer gaps or step further onto the platform (increasing their margin of error) when more afraid. Further, if the embodied perception accounts are correct in hypothesizing that changes in perceptual estimates should subserve action, then we should observe a similar magnitude of change in the performance of actions as we saw for estimating gaps when afraid.

### Method

#### Participants

Thirty (18 female, 12 male) University of Utah students with a mean eye-height of 1.62 m (*sd* = .09) participated for course credit. All participants were naïve to the purpose of the experiment, gave written informed consent, had normal or corrected-to-normal vision, and showed no deficit in stereo vision. The experimental procedure was approved by the institutional review board at the University of Utah and was in accordance with the Declaration of Helsinki.

#### Apparatus and Design

All apparati were the same as in Experiment 1. The design, height at which the gaps were viewed, and randomization of trials were the same as in Experiment 1, with two exceptions. In the current experiment, participants viewed 8 gaps (.45 m to 1.5m at .15 m intervals). In addition, participants repeated each block of trials twice. A larger number of gaps and more repetitions were presented here to more precisely and more reliably determine the point at which participants refused to step over certain gaps. The result was 92 trials (8 gap widths, 2 orders of gap presentation, 3 heights, 2 repetitions). As in Experiment 1, PPT markers (one on heel and toe of each foot) were used to accurately scale and animate the movement of virtual feet. Additionally, the position of motion-tracking markers, located on each foot, were recorded 60 times a second.

#### Procedure

After completing the informed consent, participants donned the HMD and were instructed how to perform the task. On a given trial, participants viewed a blank screen followed by the virtual height environment. Participants made the same three judgments as in Experiment 1 including the Subjective Units of Distress (SUDS) rating of fear, chance of falling, and potential for injury. Following the questions, participants were instructed to step across the gap. They were told that they should only step over the gap if they believed they could step while maintaining at least one foot on one of the platforms at all times. Participants were encouraged to perform the step as naturally as possible without jumping or hopping, but no other instructions were given to restrict how participants performed the step. The experimenter pressed a button to log the time between giving the ‘go’ signal and participants initiating the step or indicating that they did not believe they could clear the gap. If participants said they could not step across the gap, the experimenter recorded this decision and the experiment advanced to the next trial. If participants executed the step, the position of the feet via all four markers (heal and toe for each foot) was recorded throughout the step. Participants were able to see their virtual feet as they stepped. Therefore, participants received visual feedback about the success of their performance when they performed the step. After all trials, participants completed the AQ, the debriefing questionnaire, and demographic information was collected. Only five out of 30 participants correctly identified the hypothesis. One person identified the correct variables but indicated the opposite relationship. The rest indicated an influence of height or gap width on fear, or indicated that they did not know. The raw responses from each participant are available to view in the data accessible online. The whole experiment lasted about 45 minutes.

### Results

#### Measure of Fear

Fear was indexed as in Experiment 1. Scores on SUDS were person-centered so that any effect of SUDS scores was interpreted as changes in SUDS scores from individual average SUDS score. On average, participants had a positive reaction to viewing the heights of 15.65 (SD = 22.6), with a range from 0 to 100. Acrophobia scores were 35.42 (SD = 18.9), on average, with a range from 6 to 73. As in Experiment 1, Correlations between the three subjective ratings (SUDS, chance of falling, and injury from falling) were conducted for each participant. SUDS and chance of falling were highly correlated for 28 out of 30 participants (all significant rs greater than .26, all ps < .01). SUDS and perceived injury from falling were significantly correlated for 29 out of 30 participants (all significant rs greater than .22, all ps < .04). Chance of falling and perceived injury from falling were significantly correlated for 27 out of 30 participants (all significant rs greater than .21, all ps < .04). Due to these high correlations, only SUDS was used as a predictor in subsequent analyses.

### Does fear predict changes in stepping over behavior?

#### Cross-over points

To analyze differences in whether people executed an action, a cross-over point was found for each block of gap widths resulting in 6 cross-over points for each participant (twice for each height). Cross-over points were calculated by dividing the largest gap width that participants stepped over by their eye height. In this sample, average eye-height was 162.09 cm (SD = 9.6). This procedure scaled the environmental feature, the gap width, to the individual participants’ capabilities and allowed for comparisons across individuals with different abilities [27]. In the current experiment, eye-height and actual step length were positively correlated (*r =* .601, *p* < .001). Larger cross-over points indicate that participants stepped over relatively larger gap widths. In addition, for this analysis, SUDS scores were averaged across all gaps for a given height. The average cross-over point for each height is presented in Table 2.

**Table 2:** Descriptives of Stepping Behavior.

	0 m	3 m	15 m
Largest Gap Crossed:	1.27 (.21)	1.21 (.24)	1.17 (.27)
.45 m	.433 (.25)	.423 (.14)	.431 (.13)
.60 m	.404 (.11)	.409 (.13)	.413 (.14)
.75 m	.409 (.12)	.382(.14)	.393 (.15)
.90 m	.401(.12)	.371 (.16)	.367 (.17)
1.05 m	.356 (.15)	.337 (.16)	.321 (.15)
1.20 m	.298 (.15)	.271 (.14)	.266 (.12)
1.35 m	.209 (.12)	.212 (.11)	.176 (.10)
1.50 m	.194 (.12)	.14 (.07)	.115 (.06)

The mean (SD) largest gap width in meters that participants stepped over is presented for each height (column) in the first row of data. Mean (SD) margin of error is displayed for each gap width (row) and for each height (column) in meters. It is important to note that these means were averaged across participants with differing capabilities and who may have stepped across different numbers of gaps. In addition, these numbers do not reflect changes due to state or trait fear.

Differences in cross-over points were assessed using a multi-level model run in HLM 7.0. Specifically, cross-over points were regressed onto height (centered at the 3 m height), SUDS (person-centered), and AQ score (grand-mean centered). All factors were allowed to interact.

**Fig 3:**
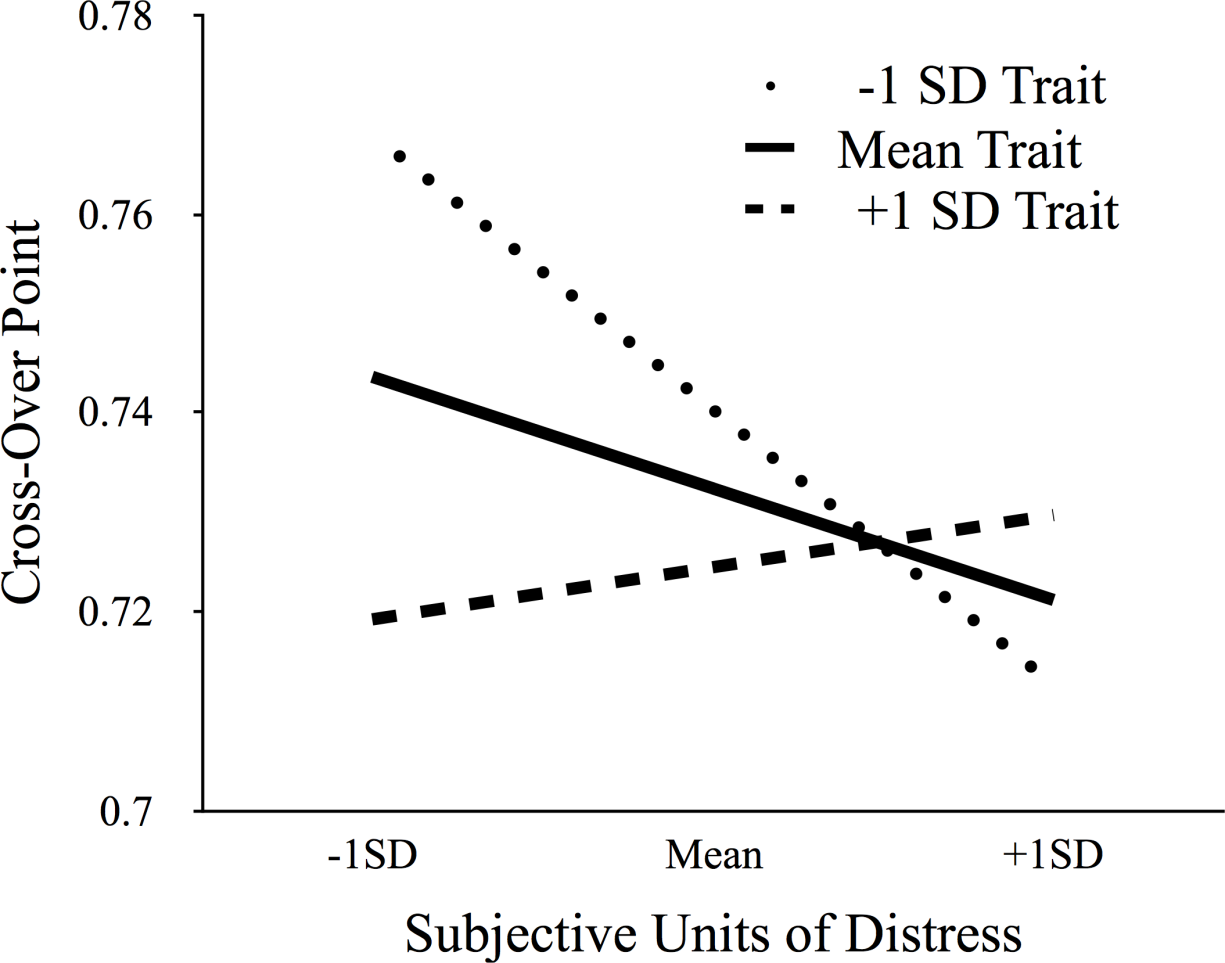
Cross-over Points as a Function of Fear. Cross-over points, or the largest gap at which participants stepped normed to eye-height, is plotted against state levels of fear (mean, +/- 1SD) for three values of trait fear (average, +/-1SD indicates high or low). Higher trait individuals were cautious, opting to step across only relatively smaller gaps. Lower trait fear individuals acted more cautiously when experiencing higher state fear than when experiencing lower state fear.

Results revealed a significant interaction between SUDS scores and AQ score on cross-over points (β = 0.00005, *p* = .029; See Fig 3). Simple slopes analyses were conducted to test for an effect of state fear on cross-over points at different levels of trait fear (+/- 1 SD). For participants with lower AQ scores (at 1 SD below the mean; AQ = 16.27), cross-over points significantly decreased as SUDS scores increased (β = -.0014, *p* < .001). That is, low trait fear individuals stepped over relatively fewer gaps as their state levels of fear increased. For participants who had higher AQ scores (at 1 SD above the mean; AQ = 52.07) the influence of SUDS scores on cross-over points was non-significant (β = .0003, *p =* 0.5). That is, high trait fear individuals did not change when they stepped as a function of their state fear. In addition, there was a significant effect of trait fear when participants experienced low state fear (β = -.0013, *p* < .001). Participants who indicated having low trait fear stepped over relatively larger gaps than participants who had high trait fear.

Overall, the results suggest that participants are more cautious in deciding when to step when they experience either situation-specific state fear or higher levels of trait fear. High trait individuals were more cautious, regardless of state fear. However, low trait individuals became more cautious as they indicated experiencing higher levels of fear. Interestingly, there was no additional influence of state fear for high-trait individuals suggesting that there may be a limit to the influence of fear on decisions to act that can be reached through either long- or short-term differences in fear.

#### Margin of error

The margin of error in the performance of the step is defined here as the distance between the near edge of the second platform and the location on the platform at which the participant’s toe landed after the step. A margin of error was calculated for each trial in which the participant actually performed the step. A larger margin of error indicates that participants stepped more cautiously because they cleared the gap by a larger amount. The average margin of error is presented in Table 2.

Differences in margin of error as a function of fear were assessed using a multi-level model run in HLM 7.0. Margin of error (in raw cm units) was regressed onto actual Gap Width (scaled to each participants eye-height and person-centered), SUDS (person-centered), Height (centered at the 3 m height), and AQ scores (grand-mean centered). All factors were allowed to interact. As in Experiment 1, gap widths were scaled to participants’ eye-height to allow for comparison across individuals with varying stepping ability. Eye-height scaled gap width had a mean of .52 (*sd* = .19, *range*: .17 to 1.04). All betas are unstandardized.

There was a significant influence of SUDS scores on margin of error (β = .0009, *p* = .03; see Fig 2B). For every one unit increase in SUDS scores, the model estimated an increase in margin of error by .0009 m. Participants cleared the gap by a larger amount on trials when they reported being more afraid (*m* = 0.346 m) compared to trials when they were less afraid (*m* = 0.385 m). On average, we saw an increase in margin of error by 3.9 cm as a function of within-person changes in state fear.

As expected there was a main effect of gap width (β = -0.459, *p* < .001) such that the margin of error decreased by an estimated .046 m when extending the gap width by 10% of the participants height. There was also a significant interaction between gap width and SUDS scores (β = -0.0035, *p* = .03) such that the influence of SUDS on margin of error decreased at wider gap widths. These effects are likely driven by participants approaching their maximum ability to step.

These results demonstrate an influence of fear on how accurately participants stepped across the gap, particularly the margin of error they allowed. Important to the goal of this work, the specific direction and magnitude of changes in performance of stepping over as a function of state and trait levels of fear was similar to that observed for the effects of fear on estimates of gap widths. Specifically, the magnitude change in the margin of error (3.9 cm) was similar in magnitude to the change in estimates of gap widths (5.3 cm) in Experiment 1 as a function of state fear.

## General Discussion

The main goal of the current paper was to determine whether fear influenced an action measure in a similar manner as estimates of spatial layout. Embodied perception accounts claim that changes in perceptual estimates, when afraid, are beneficial because they directly relate consequences of acting to the actor [1,9]. Therefore, actors should behave as if space is altered in addition to estimating space as altered. Alternatively, others have argued that biases in perceptual estimates do not necessitate a change in action because safe performance requires an accurate representation of the environment [37,41]. The current results support embodied perception claims by demonstrating an influence of fear on both how people stepped over gaps and on whether they deemed the gaps as crossable. In addition, the influence of fear on gap estimates and performance of stepping was not only in a similar direction but also to a similar magnitude. Estimates of gap widths increased by 5.3 cm as participants reported experiencing higher state fear and participants stepped farther onto the next platform by 3.9 cm when they reported experiencing higher state fear. If we had found no effect of fear on actions or one of a wildly different magnitude, then it would have been extremely unlikely that changes in perceptual estimates when afraid occur in order to reduce the likelihood of injury, as previously claimed [9].

These results could be interpreted in a variety of ways. Changes in perceived gap width due to fear could lead to changes in the performance of the action. Under this interpretation, changes in actions are due to *seeing* the gaps as larger. Recent neurological studies have found increased activation in early visual processing (within 65–90 ms after stimulus onset) in response to fearful stimuli [44] and anatomical studies in primates have found projections from amygdala to primary visual areas [45] suggesting that influences of emotion on perceptual processing may occur quite early. Alternatively, changes in both perception and action measures could be the result of some third variable associated with fear, such as an increased need to act cautiously. Under this interpretation, participants do not see the gaps as wider but respond as if it is because this is a more ‘cautious’ or less risky response. This certainly could be true for an action response, such as physically stepping over the gap where there is risk of injury if performed incorrectly. However, it is more difficult to generalize this claim to our visual-matching task given that there is no risk, or danger, with this task. The current results cannot be used to definitively conclude whether people are just overall more cautious in their responses or as evidence for perceptual changes underlying differences in action. Regardless of the underlying mechanism, we show a similar direction and magnitude of changes in the action and perception measures suggesting that the embodied perception interpretation may be plausible.

In addition, results from Experiment 1 suggest that fear of heights can influence estimates of spaces around the height, specifically gap widths. Previous research has shown fear influences estimates of heights viewed from the top but not horizontal extents on the ground [16] or heights viewed from below [23]. Heights evoke fear and are associated with a danger of falling while horizontal extents typically do not evoke fear. The current results show an influence of fear on gap widths that are raised above the ground suggesting that for an influence of fear on estimates of spatial layout to occur, there may need to be some degree of danger associated with the extent being judged.

The current findings fit into a larger body of work that suggests fear should influence action. Specifically, evolutionary approaches to emotion have long argued that one function of fear is to reduce negative consequences of goal-directed actions [46,47], and that fear may enact this function by prioritizing, maintaining, or disrupting the processing of cognitive systems in response to external stimuli [48,49]. Indeed, several studies have shown an influence of fear on different cognitive and perceptual processes. For example, emotionally laden words and objects are processed faster when afraid [50]. Contrast sensitivity is increased when afraid [51,52]. However, it is often unclear whether these perceptual changes would translate to differences in everyday actions. The current studies directly tested changes in actions when afraid and found participants altered their behavior in a manner that reduced the likelihood of injury providing direct evidence for claims about the function of fear.

The current results also inform a larger debate about the generalizability and validity of embodied perception effects (see [13,1] for contrasting perspectives). As introduced earlier, the proposed purpose of changes in perception when afraid (or in response to other non-visual changes like body size) is to inform future actions. The embodied perception approach has been criticized, in part, because the size of the influence of non-visual factors on perceptual measures was believed to be too small, too large, or wildly mis-calibrated to any potential change in action performance [13]. We found evidence for a similar effect size of fear on estimates of gap widths and stepping over behavior suggesting that these response measures may be related and possibly calibrated to one another. However, more work needs to be conducted to investigate whether these hypotheses hold true and also whether other manipulations (i.e., changes to body size) similarly alter perceptual estimates and performance of actions.

In the current set of experiments, we made specific design choices in an attempt to reduce the influence of task demands and the potential for experimenter bias, which have also been criticized by opponents of embodied perception in the past [53,13]. One potential concern is that our results may be due to task demands, or our participants intuiting the hypothesis of the study and altering their responses accordingly. In the current experiments, we attempted to reduce these possibilities by measuring individual differences, in terms of trait or state fear, and utilizing a different set of subjects for the perception and action measures. In this manner, participants were unaware of the predictors and questions being addressed, which should make it more difficult for them to consciously bias their responses. Admittedly, participants rated state fear on every trial but it is unclear why this would have created larger demands for low trait fear individuals than high trait fear individuals, as the effect of state fear on cross-over points was only present for those with low trait fear. An additional concern when conducting psychological experiments is the potential influence of an experimenter unconsciously (or consciously) altering their behavior and biasing responses. In previous studies on the influence of fear on height perception, the experimenter acted as one end of the reference extent during the matching task. In response to worries about potential biasing of results by experimenter involvement in the estimation task, attempts have been made recently in real-world experiments to reduce the involvement of the experimenter [54]. Similarly, in the current experiments, participants manipulated the length of the reference extent within the virtual environment and all data was saved digitally—thus excluding the experimenter from the data collection process.

In addition, when attempting to understand the influence of emotion on perceptual processes, and even other cognitive tasks, it is important to strike a balance between experimental control and ecological validity. We believe virtual reality is an excellent tool in this regard. Using virtual reality also allowed us to present multiple heights and gap widths to participants with relative ease. Previous studies of fear on height perception often employed only one or two measures of state fear leading to difficulty in disentangling state and trait differences [7,8]. Finally, virtual reality allowed participants to perform actions that would be dangerous in the real world without the threat of any real danger. Despite no real danger, work by Meehan, Insko, Whitton, and Brooks [55] and Seinfeld et al. [56] have found physiological measures of fear increased when viewing virtual heights, suggesting that participants react to heights in virtual environments as if they were real and that virtual environments are a reliable method for inducing emotional state. Thus, virtual reality serves as a great tool to manipulate the perception of danger without unnecessarily exposing participants to actual danger.

## Conclusions

Three important findings were reported in the current research. First, estimates of gap widths increased with increases in state fear suggesting that fear can influence the perception of spatial properties around heights. Second, participants were less willing to step over gaps when they experienced more state or trait fear. They also stepped farther over gaps when experiencing higher state fear. Finally, the effect size of the influence of fear on estimates of gap widths and stepping behavior were remarkably similar in both direction and magnitude. A change in stepping behavior due to fear that is similar to a change in estimates of gap widths supports the functional claim of embodied perception that changes in the perception of spatial layout when afraid should lead to differences in actions.
